# Analysis of changes in tibial torsion angle on open-wedge high tibial osteotomy depending on the osteotomy level

**DOI:** 10.1186/s43019-021-00127-x

**Published:** 2022-03-28

**Authors:** In-Soo Song, Junhan Kwon

**Affiliations:** Department of Orthopedic Surgery, Daejeon Sun Hospital, 29, Mokjung-ro, Jung-gu, Daejeon, Republic of Korea

**Keywords:** Knee, Kellgren–Lawrence grade 3 osteoarthritis, Tibial torsion angle, Open-wedge high tibial osteotomy, High level osteotomy

## Abstract

**Purpose:**

This study evaluated the tibial torsional angle changes of 72 knees before and after open-wedge high tibial osteotomy (OWHTO) and compared the results according to the osteotomy level.

**Materials and methods:**

Seventy patients (72 knees) with Kellgren–Lawrence grade 3 underwent OWHTO. Demographic data, operation procedures, and measurement of mechanical tibiofemoral angle (mTFA), anatomical tibiofemoral angle (aTFA), tibial torsional angle (TTA), and pre- and postoperative Lysholm and International Knee Documentation Committee (IKDC) scores were obtained. The authors analyzed TTA changes between 30 knees with high-level osteotomy (group A) and 42 knees with low-level osteotomy (group B).

**Results:**

The changes of TTAs in the subjects of 72 knees went from 29.26 ± 5.6° preoperative mean to 25.36 ± 6.4° postoperative mean (*p* = 0.032). The postoperative TTAs of group A (mean 27.4 ± 4.8°) and B (mean 25.7 ± 4.9°) were statistically significant (*p* < 0.01). Preoperative Lysholm and IKDC scores of 72 knees had means of 49.1 ± 3.5 and 49.0 ± 15.2, respectively, and postoperative means of 85.7 ± 8.56 and 78.0 ± 17.6, respectively, which were statistically significant (*p* < 0.01).

**Conclusions:**

Changes of TTA with internal rotation of distal tibia were observed following OWHTO. High-level osteotomy on the proximal tibia’s lateral cortex had less internal rotation of the distal tibia than low-level osteotomy.

## Introduction

Medial open-wedge high tibial osteotomy (OWHTO) is a widely recognized surgical treatment option for medial unicompartmental osteoarthritis accompanied with varus malalignment [1–6]. Achieving accurate alignment correction following OWHTO is detrimental to successful clinical outcomes. However, surgeons have previously focused on coronal plane correction and disregarded any possible changes on either the sagittal or axial plane during OWHTO [7]. Moreover, to date, OWHTO-associated rotational malalignment has not been appropriately addressed, and limited studies have discussed changes in the axial tibial rotation following OWHTO [8, 9]. However, studies demonstrating the occurrence of unintentional secondary distal tibial rotational changes during OWHTO have recently emerged [10]. As the internal rotation of the distal tibia with the proximal fragment commonly occur after OWHTO [11], excessive changes in the tibial torsional angle (TTA) may adversely interrupt the bone union after osteotomy and affect clinical results, including gait pattern and patellar tracking. Therefore, such changes could eventually lead to an increased risk of patellofemoral osteoarthritis [12].

**Fig. 1:**
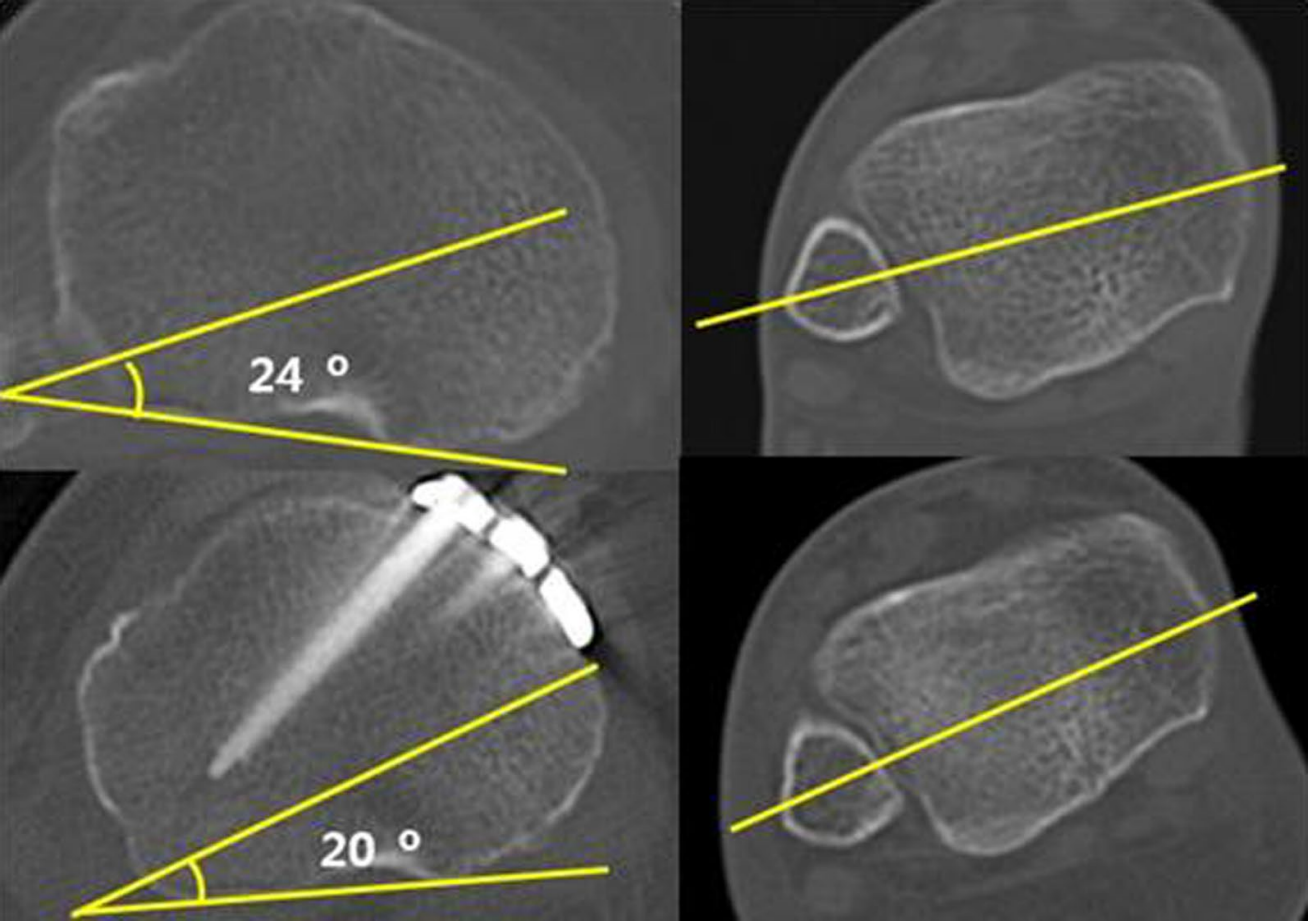
A change in tibial torsion angle (TTA) pre- and postoperation on axial computed tomography was measured. TTA, an angle formed by the axis through the posterior tangent of the widest proximal tibial condyles to the transverse axis that bisects the anteroposterior (AP) diameter of the lower end of the tibia and passes through the anterior half of the lateral malleolus in the axial plane, using computed tomography (CT)

Standard plain radiographs have failed to successfully evaluate such change in rotation [9]. This study measured the TTA, an angle formed by the axis through the posterior tangent of the widest proximal tibial condyles to the transverse axis that bisects the anteroposterior (AP) diameter of the lower end of the tibia and passes through the anterior half of the lateral malleolus in the axial plane, using computed tomography (CT) (Fig. 1) [13]. CT imaging of all knees were obtained from 2 mm CT scan cuts from both proximal and distal tibia. The decrease in the TTA shows an increased internal rotation of the tibia [14].

This study aims to assess the TTA changes before and after OWHTO. TTA changes were verified and divided into the lateral cortical osteotomy lines above the fibula’s tip and those below the fibula’s tip. Based on prior clinical observations, it was hypothesized that OWHTO could change the TTA result in the distal tibia’s internal rotation in relation to the proximal tibia.

## Materials and methods

The study commenced after the approval of the Institutional Review Board. CT images were evaluated in 70 patients (72 knees) with varus malalignments of Kellgren–Lawrence grade 3 with varus medial compartmental lesion. Patients were retrospectively enrolled and underwent biplane medial OWHTO between June 2016 and March 2019. An orthopedic surgeon performed the OWHTO. The presence of meniscal tears as operative indications was not considered. This study’s ineligibility criteria were Kellgren–Lawrence grade 3 with concomitant lateral compartmental OA, Kellgren–Lawrence grade 3 with presence of normal aligned lower extremity with proximal tibia vara, rheumatoid arthritis, posttraumatic arthritis, infectious arthritis, peroneal nerve injury, lateral hinge fracture, nonunion of the osteotomy site, and revision high tibial osteotomy (HTO). The mean age of the sample population was 60.8 ± 5.1 years. The sample was composed of 48 female and 22 male participants, with a mean BMI of 25.87 ± 5.8 kg/m^2^. The average follow-up period was 26.3 months, and the degrees of lower extremity deformity of the patients showed varus 6.1 ± 2.2° (Table 1). All knees were radiographically and clinically assessed before and after the surgery.

**Table 1:** Patient demographics

Characteristic (*n* = 72)	Mean (range) or *N*
Age, years	60.8 ± 5.1 (48–71)
Sex, *n*	Male, 8; Female, 38
BMI*, kg/m^2^	25.87 ± 5.8 (20.45–31.25)
Follow-up, months	26.3 (3–30)
Kellgren–Lawrence grade	All Grade III
Degrees of lower extremity deformity	Varus 6.1 ± 2.2° (Varus 2.5°–11°)

*BMI* body mass index

### Surgical technique

Preoperative planning was done through standing anteroposterior (AP) long bone, standing AP, and lateral knee joint radiograph evaluation. The osteotomy site, correction angle, and osteotomy gap size were measured using the method described by Miniaci et al. [15]. The articular cartilage status, menisci, and cruciate ligaments underwent a diagnostic arthroscopy assessment afterward. In the process, meniscal procedures, including repair or meniscectomy, were performed as necessary.

**Fig. 2:**
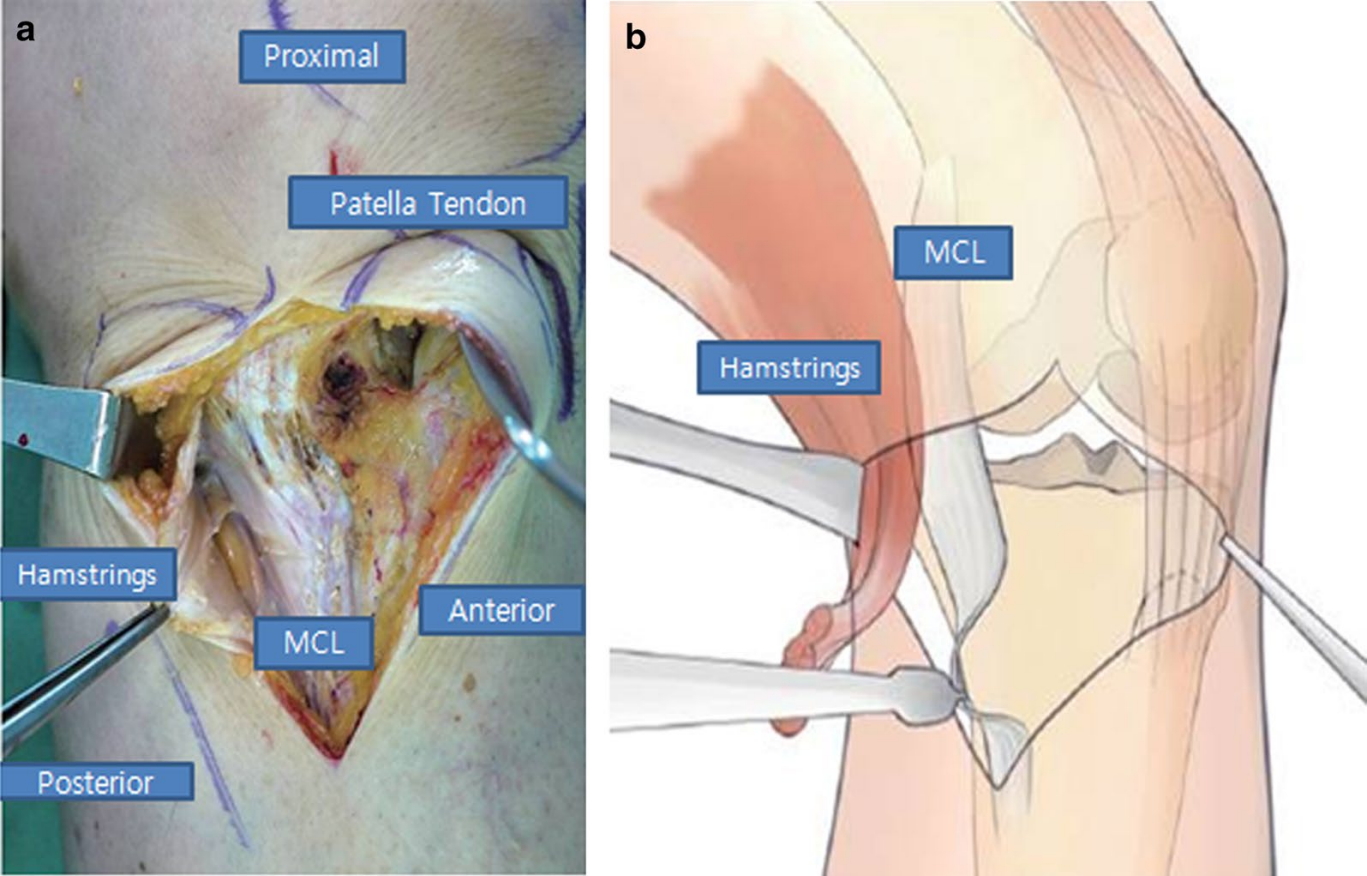
**a **The hamstring tendon was cautiously exposed after facial incision and vertical release of the distal tendon insertion’s detachment. **b** The medial collateral ligament’s distal fibers were exposed and released distally between the exposed medial collateral ligament and the tibia’s posteromedial side

**Fig. 3:**
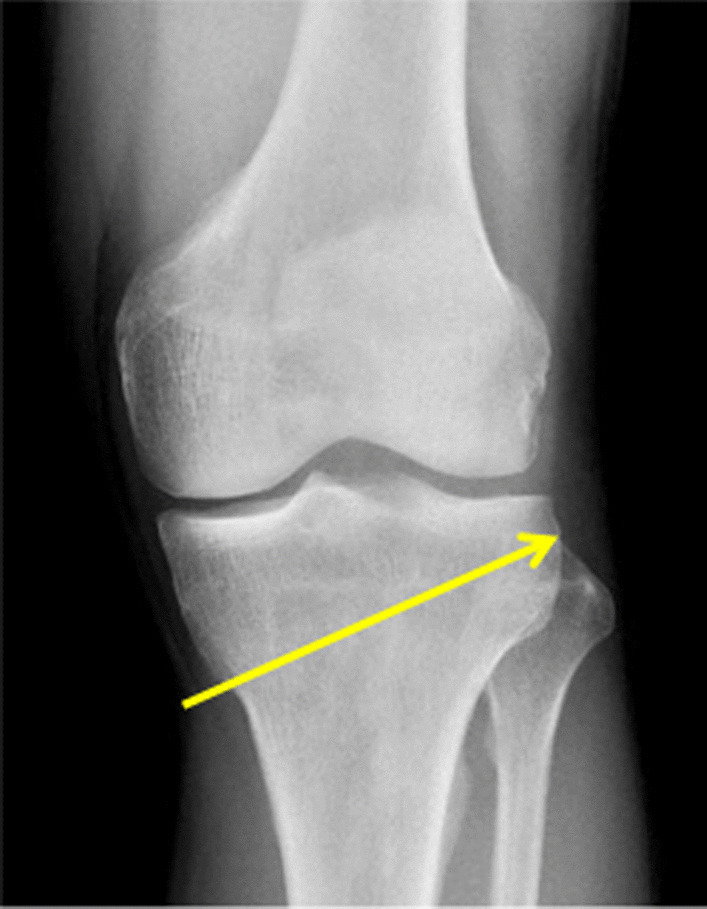
The targeted osteotomy line around the tip of the fibular head was routinely made on all operations. The osteotomy line above the tip of the fibula can lead to nonunion or condyle fracture

An approximately 7 cm anteromedial vertical skin incision was made approximately two-finger breaths medial to the tibial tuberosity. A sharp dissection exposed the sartorial fascia, and the pes anserinus tendon was distally retracted. The medial collateral ligament’s distal fibers were accessed and distally released. A Barret retractor was inserted to protect the neurovascular structure between the exposed medial collateral ligament and the tibia’s posteromedial side. Afterward, a vertical release through the fascia of the medial collateral ligament, pes anserinus, and posteromedial capsule (Fig. 2a, b) without incision was performed. Two 2.0 K-wires were used as guide wires. The first 2.0 K-wire was under fluoroscopic control and inserted into the fibula tip anteriorly, and the second 2.0 K-wire was inserted parallel to the tibial slope. To avoid the proximal migration of the osteotomy into the joint, osteotomy was performed immediately distal to the guidewire. The biplane osteotomy’s oblique osteotomy began from the medial tibial cortex and to the level around the fibular head’s tip (Fig. 3). Osteotomies advanced to approximately 1 cm medial of lateral cortex without breakage using a controlled fluoroscopic guidance system. The biplane osteotomy’s vertical osteotomy was performed on the tibial tuberosity’s posterior aspect to avoid traumatizing the patellar tendon’s bony insertion.

A gap spreader was used to open the osteotomy site, and then a metal block was inserted. The osteotomy site was gradually opened with caution to the planned width using chisels to avoid lateral cortical damage. The osteotomy site’s stability was maintained using a fixed angle plate with a locking screw (Viper Fix^®^: SI Medisys, Goyang, Republic of Korea, *n* = 72). The fixation was performed on a partially flexed knee joint.

### Radiological evaluation

Radiological evaluation was performed using weight-bearing AP, knee joint lateral views, and CT scans of the lower extremities [13, 16, 17]. Radiographs were taken the day before and 3 months after the operation. Moreover, CT scans were done 2 weeks after the operation. In this study, the radiological parameters accounted for were the mechanical tibiofemoral angle (mTFA), the anatomical tibiofemoral angle (aTFA), and the TTA (Fig. 1).

**Fig. 4:**
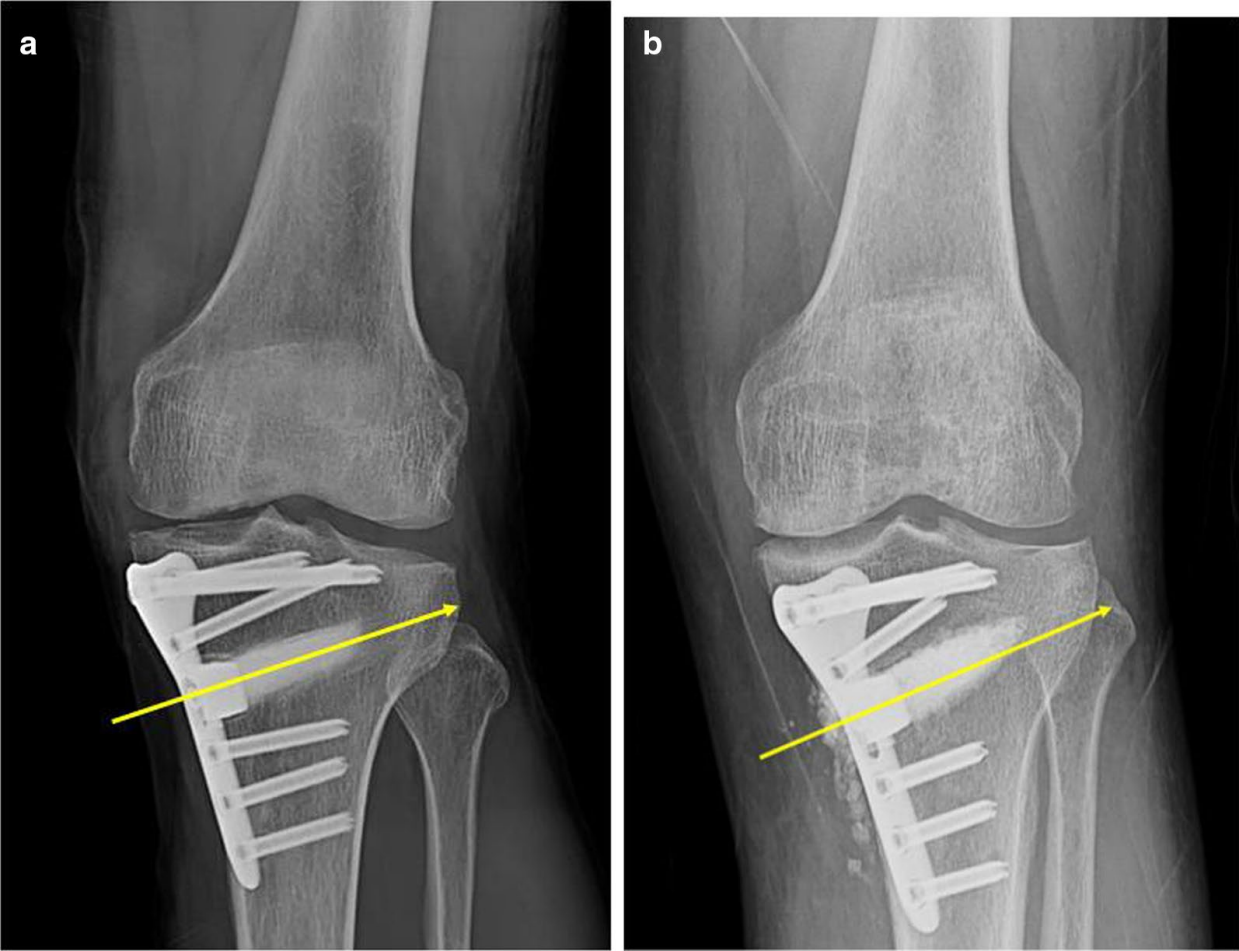
Immediate postoperative anteroposterior (AP) view radiography of the knee after open-wedge high tibial osteotomy (OWHTO). Patients were categorized into two groups: **a** A lateral cortical osteotomy line above the tip of the fibula and **b** a lateral cortical osteotomy line below the tip of the fibula

Based on the radiographs, the patients were divided into two groups: one group (group A) with the lateral cortical osteotomy lines above the tip of the fibula (aTTA, 30 knees) and another (group B) with the lateral cortical osteotomy lines below the tip of the fibula (bTTA, 42 knees Fig. 4a, b). Clinical analysis was performed through the comparison of the preoperative and postoperative Lysholm and International Knee Documentation Committee (IKDC) scores. The collective data was gathered using Microsoft Excel 2010 version (Microsoft Corp., Redmond, WA, USA) and presented as mean ± standard deviation. Pre- and postoperative comparisons of the mTFA, aTFA, and TTA were made using paired *t*-tests, and Student’s *t*-test was used to compare aTTA and bTTA. All obtained statistical data were analyzed using SPSS 20.0 (SPSS Inc., IL, USA).

## Results

Three different individuals measured the radiographic parameters. Measurements were repeated once by each examiner, and mean values between the two were calculated to minimize measurement errors. Interobserver reliability assessment was performed using Winner’s criteria [18]. Reliability was categorized by intraclass correlation coefficient as absent-to-poor (0.00–0.24), low (0.25–0.49), fair-to-moderate (0.50–0.69), good (0.70–0.89), or excellent (0.90–1.0). Interobserver reliability was 0.94.

The mTFAs were corrected from preoperative mean varus 6.1 ± 2.2° to postoperative mean valgus 1.38 ± 2.7°, showing statistical significance (*p* = 0.021). The mean preoperative and postoperative aTFAs were varus 0.26 ± 1.4° and valgus 6.53 ± 1.9°, respectively, showing statistical significance (*p* = 0.016). Except for four knees, most of the cases were internally rotated postoperatively. The changes of TTAs in 72 knees were from preoperative mean 29.26 ± 5.6° to postoperative mean 25.36 ± 6.4°, showing statistical significance (*p* = 0.032).

The changes of TTA in group A (30 knees) went from preoperative mean 29.6 ± 4.4° to postoperative mean 27.4 ± 4.8°, showing statistical significance (*p* = 0.041). The changes of TTA in group B (42 knees) went from preoperative mean 29.6 ± 5.3° to postoperative mean 25.7 ± 4.9°, showing statistical significance (*p* = 0.024; Table 2). The difference between preoperative aTTAs and preoperative bTTAs was not statistically significant (*p* = 0.741), but the difference between postoperative aTTA (27.4 ± 4.8°**)** and postoperative bTTA (25.7 ± 4.9°) was statistically significant (*p* < 0.01; Table 3). The mean preoperative and postoperative Lysholm scores of 72 knees were 49.1 ± 3.5 and 85.7 ± 8.6, respectively, and they were statistically significant (*p* < 0.01). The mean preoperative and postoperative IKDC scores of 72 knees were 49.0 ± 15.2 and 78.0 ± 17.6, respectively, and they were statistically significant (*p* < 0.01; Table 4). However, postoperative Lysholm scores of aTTAs and bTTAs (87.4 ± 8.2 and 83.1 ± 9.6, respectively) showed no statistical significance (*p* = 0.917) (Table 5). Also, postoperative IKDC scores of aTTAs and bTTAs (82.0 ± 12.2 and 75.2 ± 16.8, respectively) showed no statistical significance (*p* = 0.893) (Table 6).

**Table 2:** Radiological results

Variable	Preoperation	Postoperation	*p*-Value
mTFA	6.1° ± 2.2° Varus	1.38° ± 2.7° Valgus	0.021
aTFA	0.26° ± 1.4° Varus	6.53° ± 1.9° Valgus	0.016
TTA	29.26° ± 5.6°	25.36° ± 6.4°	0.032
aTTA (*n* = 30)	29.6° ± 4.4°	27.4° ± 4.8°	0.041
bTTA (*n* = 42)	29.3° ± 5.3°	25.7° ± 4.9°	≤ 0.01

*aTTA* lateral cortical osteotomy above the tip of the fibula, *bTTA* lateral cortical osteotomy below the tip of the fibula

**Table 3:** Comparison of aTTAs and bTTAs

	aTTA (*n* = 30)	bTTA (*n* = 42)	*p*-Value
Preoperative TTA	29.6° ± 4.4°	29.3° ± 5.3°	0.741
Postoperative TTA	27.4° ± 4.8°	25.7° ± 4.9°	< 0.01
*p*-Value	0.041	< 0.01

**Table 4:** Clinical results

Variable	Preoperation	Postoperation	*p*-Value
Lysholm (*n* = 72)	49.1 ± 3.5	85.7 ± 8.6	< 0.01
IKDC (*n* = 72)	49.1 ± 15.2	78.0 ± 17.6	< 0.01

*IKDC* International Knee Documentation Committee

**Table 5:** Comparison of Lysholm scores of a TTAs and bTTAs

Variable	aTTA (*n* = 30)	bTTA (= 42)	*p*-Value
Preoperative Lysholm	53.6 ± 2.7	50.7 ± 3.4	0.864
Postoperative Lysholm	87.4 ± 8.2	83.1 ± 9.6	0.917

*IKDC* International Knee Documentation Committee

**Table 6:** Comparison of IKDC scores of aTTAs and bTTAs

Variable	aTTA (*n* = 30)	bTTA (= 42)	*p*-Value
Preoperative IKDC	47.5 ± 12.4	49.4 ± 13.8	0.902
Postoperative IKDC	82.0 ± 12.2	75.2 ± 16.8	0.893

*IKDC* International Knee Documentation Committee

## Discussion

The most significant finding of this study was the noticeable change in TTAs following biplane medial OWHTO. According to Jacobi et al. [10], possible factors that influence the change in TTAs during OWHTO are variable stability of the lateral hinge, variable tension of posterior soft tissues, and other unidentified reasons. The presence of osteoarthritis in the tibiofemoral joint was mostly a detrimental factor, which could lead to posterior soft tissue contracture and contribute to the decreased tibial rotation. The extent of correction performed was another parameter affecting tibial rotation change. The larger the extent of the correction, the higher the likelihood of changes for tibial rotation. For patients with concomitant anterior cruciate ligament (ACL) degeneration, small correction changes will derive an extensive change in TTA. The ACL resists not only the motions of anterior tibial translation but also those of internal tibial rotation. The other factor that could have influenced the results was the position of the knee, whether flexion or extension, during the procedure, as changes to the internal rotation were more pronounced in extension than in flexion.

Hinterwimmer et al. [11] stated that two anatomical conditions might lead to a significant tibial rotation. First, the medial soft tissue structures may play a role leading to a tibial rotation. In OWHTO, the pes anserinus is posteromedially retracted, and the insertion of the semitendinosus tendon is maintained distal to the osteotomy. ‟During OWHTO, when the osteotomy site is opened and the osteotomy is performed, these tendons’ increased tension may lead to an internal tibial rotation.”

‟Second, this is due to the complex three-dimensional anatomy of the proximal tibia during osteotomy.” ‟The operation’s objective is to redistribute the force on the tibiofemoral joint with valgisation of the distal tibia.” ‟Moreover, lateral column preservation is crucial to the primary stability of the osteotomy gap.” ‟Proper preparation of soft tissue at the posterior aspect of the tibia is required to preserve the lateral column.” However, concerns regarding muscle and neurovascular injuries could lead to improper preparation [11]. Therefore, the posterior and posterior-lateral cortex’s osteotomy is not performed with ease because it can lead to a misguided hinge axis direction [19].The variation in distinct hinge axis location and direction may influence the distal tibial rotation [20].

‟There are numerous methods to assess the tibial torsional angle, including clinical, anthropometric, and cadaveric skeletal measurement and imaging techniques, including CT, fluoroscopy, magnetic resonance imaging (MRI), and ultrasonography.” ‟Nowadays, tibial torsion assessment using a CT scan is considered an accurate and reliable golden method” [13]. The examiner has to calculate the angle between the tibia’s proximal and distal articular axis in the transverse computerized tomograms [17] to assess tibial torsion. Decreased TTA can be interpreted as the internal rotation of the tibia’s distal fragment, and increased TTA can be interpreted as external rotation [16].

Because the tibia is a three-dimensional triangular shape, if the lateral end of osteotomy was above the fibula tip, it will yield a larger surface area than below the fibula’s tip, leading to a significant difference inTTA. Compared with the larger surface area, the angle change will be greater in the smaller surface area, even if the same amount of rotation is applied. As stated above, the same result is observed in this study. By performing lateral osteotomy above the fibula’s tip, the tibia’s distal fragment was significantly less internally rotated than when performing lateral osteotomy below the fibula’s tip.

With regards to clinical scores, both mean preoperative and postoperative Lysholm and IKDC scores of 72 knees showed statistically significant differences. However, clinical scores of posteoperative aTTAs and bTTAs did not show the statistically significant differences. Even though the clinical scores of postoperative aTTAs and bTTAs did not show significant difference compared with bTTAs, better clinical results were shown in aTTAs. This can be interpreted as the slight changes of such rotations: patients’ clinical symptoms have been improved due to the correction of alignment, which transfers the mechanical loading from the affected medial compartment to the healthy lateral compartment [1–6].

There are numerous factors that may influence the internal rotation of distal tibia in this study. Improper externally rotated ankle position during osteotomy and plate fixation, soft tissue dissection and releasement, and the angle between the oblique and vertical osteotomy plane are thought to be critical.

This limitations of this study should be noted, such as the very small number of patients enrolled in this study. Moreover, although education of patients regarding the lower limb position during radiographic evaluations was done beforehand, the measurements evaluated using radiographs and CT images are two-dimensional, and the influence of limb position and scanning direction on the collected data cannot be ignored.

## Conclusion

The open-wedge high tibial osteotomy showed statistically significant changes in the tibial torsional angle, such as the distal tibia’s internal rotation regarding the proximal tibia. A lateral osteotomy level higher than fibular tip on the proximal tibia’s lateral cortex presents significantly lower chances of internal rotation of the distal tibia.

## Data Availability

The datasets generated during and/or analyzed during the current study are available from the corresponding author on reasonable request.

## Abbreviations

OWHTO: Open-wedge high tibial osteotomy
mTFA: Mechanical tibiofemoral angle
aTFA: Anatomical tibiofemoral angle
TTA: Tibial torsional angle
IKDC: International Knee Documentation Committee
AP: Anteroposterior
CT: Computed tomography
OA: Osteoarthritis
HTO: High tibial osteotomy
ACL: Anterior cruciate ligament
MRI: Magnetic resonance imaging
